# Case Report: Progression of a Silent Corticotroph Tumor to an Aggressive Secreting Corticotroph Tumor, Treated by Temozolomide. Changes in the Clinic, the Pathology, and the β-Catenin and α-SMA Expression

**DOI:** 10.3389/fendo.2022.870172

**Published:** 2022-07-19

**Authors:** Gianina Demarchi, Sofía Perrone, Gaela Esper Romero, Cristian De Bonis, Juan Pablo Casasco, Gustavo Sevlever, Silvia Ines Berner, Carolina Cristina

**Affiliations:** ^1^ Centro de Investigaciones Básicas y Aplicadas (CIBA), Universidad Nacional del Noroeste de la Provincia de Buenos Aires (UNNOBA), Junín, Buenos Aires, Argentina; ^2^ Centro de Investigaciones y Transferencia del Noroeste de la Provincia de Buenos Aires (CITNOBA, Universidad Nacional del Noroeste de la provincia de Buenos Aires (UNNOBA)-Consejo Nacional de Investigaciones Científicas y Técnicas (CONICET)) Pergamino, Buenos Aires, Argentina; ^3^ Servicio de Neurocirugía, Clínica Santa Isabel, Buenos Aires, Argentina; ^4^ Departamento de Neuropatología, Instituto FLENI, Buenos Aires, Argentina

**Keywords:** ACTH, tumor transformation, β-Catenin, temozolomide, corticotroph tumor, vasculature

## Abstract

Clinically silent corticotroph tumors are usually macroadenomas that comprise 20% of ACTH tumors. They frequently progress to aggressive tumors with high recurrence, invasiveness, and on rare occasions, they may become hormonally active causing Cushing’s disease. Trustable biomarkers that can predict their aggressive course, as well as their response to traditional or new therapies, are paramount. Aberrant β-Catenin expression and localization have been proposed as responsible for several malignancies including pituitary tumors. Nevertheless, the role of β-Catenin in the aggressive transformation of silent corticotropinomas and their response to Temozolomide salvage treatment have not been explored yet. In this work, we present a case of a silent corticotroph tumor that invaded cavernous sinus and compressed optic chiasm and, after a first total resection and tumor remission it recurred six years later as an aggressive ACTH-secreting tumor. This lesion grew with carotid compromise and caused Cushing’s signs. It required multiple medical treatments including Cabergoline, Ketoconazole, TMZ, and radiotherapy. Besides, other two surgeries were needed until it could be controlled. Interestingly, we found α-SMA vascular area reduction and differential β-Catenin cell localization in the more aggressive tumor stages characterized by high Ki-67 indexes and p53 expression. Our results may indicate a role of angiogenesis and β-Catenin trigged events in the pituitary tumor progression, which could in turn affect the response to TMZ and/or conventional treatments. These molecular findings in this unusual case could be useful for future management of aggressive pituitary tumors.

## Introduction

Adrenocorticotropic hormone (ACTH)-secreting pituitary tumors that cause Cushing’s disease account for 5% to 10% of pituitary adenomas. Silent corticotroph adenomas are an uncommon subgroup of nonfunctioning pituitary tumors (NFPAs) (1) characterized by no biochemical or clinical evidence of Cushing’s disease (2, 3). They are generally diagnosed after surgery by positive immunostaining for ACTH (4). They represent approximately 20% of all corticotroph adenomas and approximately 5% of NFPAs (3, 5).

These silent pituitary tumors are usually macroadenomas and have a more aggressive behavior with a higher chance of hemorrhage and invasion of anatomical critical structures (6). On rare occasions, they transform to active Cushing’s disease, with high serum ACTH levels (5). Moreover, for silent corticotroph adenomas, there has been reported a high risk of recurrence. For those patients whose tumor recurs or progresses, treatment includes reoperation, combined medical approaches, and/or stereotactic radiotherapy (7–9). There is growing experience in the use of TMZ to treat these aggressive tumors and many studies that relate to its effectiveness with the expression of MGMT and other methyltransferases (10, 11).

The complexity of silent corticotroph tumors challenges their appropriate diagnosis and clinical management. Identification of markers that can predict the aggressive progression of pituitary tumors may be useful in the treatment strategy and could be of assistance in preventing recurrence.

β-Catenin is essential for embryonic development and is required for cell renewal/regeneration in adult life. β-Catenin-mediated signaling has been associated with tumor-initiating cells in multiple malignancies (12). Its activation promotes cell proliferation and supports tumor growth. Aberrant expression of β-Catenin has a role in cellular transformation, tissue invasion, and metastasis (13). Moreover, β-Catenin contributes to the protumoral angiogenic process in different cancers (14–16).

The present case is a rare silent corticotroph tumor that evolved into an aggressive ACTH secreting tumor and required repeated surgeries as well as combined medical treatment including Temozolomide (TMZ). Across the follow-up, pathology studies showed cellular abnormalities, increased Ki-67 proliferation index, variable p53 immunostaining, and necrotic areas. Molecular biology analysis showed singular changes in tumor vascularity as well as in the expression and localization of the β-CATENIN protein.

Our analysis and results contribute to understanding the processes that occur along different transforming stages in the aggressive pituitary tumor evolution. They provide promising data that could be helpful in new treatment design for aggressive and resistant pituitary tumor management.

## Materials and Methods

### Pathology and Tumor Samples

Pre and post-surgical imaging, pathology, and diagnoses were carried out at the pathological service. Routine technique and immunohistochemistry were performed. Data are summarized in **Table 1**.

**Table 1:** Clinical, biochemical, imaging, and pathological findings during the clinical transformation from silent corticotroph tumor to aggressive ACTH-secreting tumor.

	Course of the desease												
	2008		2009	2014		2015		2016	2017	2018	2019	2020	2021
	Pre surgery	Post surgery		Pre surgery	Post surgery	Pre surgery	Post surgery						
**Cushing symptoms**	Absent	Absent	Absent	Increased body weight, centripetal obesity, high blood pressure and diabetic symptoms	Ameliorated clinical symptoms	Increased body weight, centripetal obesity	Improvements in patient´s clinical features	Stabilized Cushing disease					
**Biochemistry**													
**PRL**NV: 1.8-20.3 ng/ml	7	5.6	10	11	7.0	7.0	5.6	5.2	6.9	3.4	0.8	0.4	0.1
**TSH**NV: 0.27-5.20uIU/ml	2.45	1.25	2.4	2.2	0.79	0.74	1.5	1.07	0.54	1.5	2.43	0.89	1.22
**fT4**NV: 0.61-1.39uIU/ml	0.85	1.0	ND	ND	ND	0.99	ND	1.25	ND	ND	ND	ND	ND
**LH**NV: PMF = 15.9-54mIU/ml	12.2	7.6	7.4	10	11.2	6.9	6.5	5.8	5.9	5	4.3	3.9	3.8
**FSH**NV: PMF = 23-116mIU/ml	7.9	8.2	9.4	9.2	9.8	6.0	7.0	7.2	7	7.3	7.5	7.4	7.2
**ACTH**NV: up to 46 pg/ml	33	ND	ND	175	158	215	87	117	74	110	120	126	171
**Plasmatic Cortisol**NV: 6.2- 23 ug/dl	14.8	11.1	7.9	36	33.2	36.2	16.7	14.4	18	36	31	27.3	19.1
**Urinary Cortisol**NV: 20-90 ug/24h	ND	ND	ND	436	338	413	91.2	129	92	73	47	123	176
**Salivary Cortisol**NV: up to 1.5 ug/dl	ND	ND	ND	ND	ND	3.78	2.34	ND	1.81	0.37	0.73	ND	3.79
**GH**NV: 0-5ng/ml	0.23	0.17	0.1	0.17	0.17	0.15	0.19	0.17	0.1	ND	0.3	0.23	0.27
**IGF1**NV: 61-239ng/ml	51.7	42.2	104	ND	70	36	ND	55.8	ND	60.3	ND	ND	42.3
**Tumor**													
**MRI**	Sellar; para and suprasellar lesion. Cavernus sinus invasion. Optic chiasm involvement	Total resection	–	Giant tumor mass. Nasal invasion, cavernous sinuses infiltration. Carotid arteries compromise	Partial resection	–	Partial resection	Tumor remain stable after the last surgery					
**Size** (mm)	31x28x24	–	–	54.3x32x23	21.1X19.3X10.9	29x31x23	25.6X23X18.9
**Diagnostic**	–	Silent corticotrophtumor	–	–	**Aggressive** ACTH-secreting pituitary tumor	–	ACTH-secreting pituitary tumor	–					
**Pathology**													
**ACTH**		+			–		+++						
**Ki-67**		<1%			9%		4%
**Mitosis**		–			+		<1%
**p53**		ND			+		<1%
**MGMT**		ND			Negative		Negative
**β-CATENIN**		ND			High in membranes/low in cytoplasms		Low inmembranes/high in cytoplasms
**α-SMA**		Hypervascularization			Low vascular area		Low vascular area

ND, Not determined; NV, Normal values; PMF, Post menopausal female; fT4, freeT4 hormone.

## Methods

### Immunohistochemistry (IHC)

Immunoperoxydase technique was used for β-Catenin and α-SMA detection. We proceeded as we previously described (17). The following primary antibodies were used: rabbit polyclonal anti-β-Catenin (#06-734, Millipore, dilution 1/300), and rabbit monoclonal anti-α-SMA (#19245, Cell Signaling, dilution 1/300). Replacement of the primary antibody with PBS was used as a negative control

ACTH, Ki-67, p53, and MGMT were performed by the BOND MAX TM automatic system (Leica Biosystems) in our laboratory of Neuropathology.

### Quantification

For β-Catenin expression, cell membranes, cytoplasms, and nuclei were counted, relativized to total nuclei, and expressed as percentages. At least three images of every surgery tissue were analyzed. For the angiogenic marker α-SMA, pictures of the total tumor samples were taken. The vascular area was determined by quantifying the cumulative area occupied by α-SMA (+) vessels in relation to the total area (α-SMA (+) area/total area). Microvascular density (MVD) was determined by counting the number of α-SMA (+) vessels per millimetre^2^, and the size of vessels is presented as the average total vessels size.

## Case Description

### Clinical Presentation

We analyzed the case of a 65-year-old-woman who in 2008 presented with neuro-ophthalmologic signs such as acute visual field defects, bitemporal hemianopsia, and headache. MRI showed a sellar, supra, and para sellar lesion with cavernous sinus invasion and optic chiasm involvement; with hemorrhagic zones compatible with a pituitary tumor. The woman also related gain of weight and tiredness. Laboratory analysis showed hypopituitarism and hypocortisolism that required hormone replacement.

**Figure 1:**
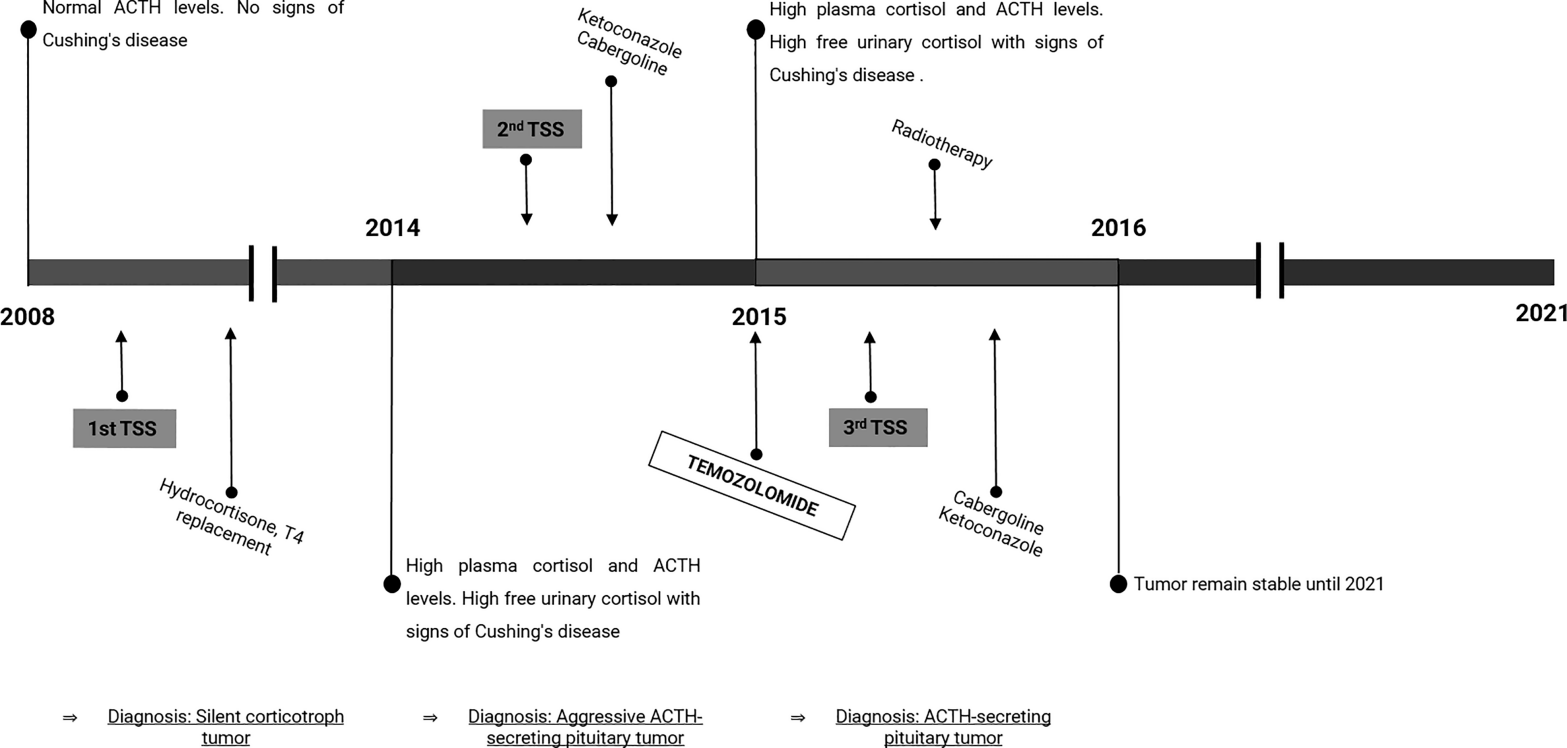
Timeline of disease progression and treatment. The scheme represents the course of the disease along time, including the diagnosis, hormone levels, and treatments. TSS, Transsphenoidal surgery; T4, thyroxine.

**Figure 2:**
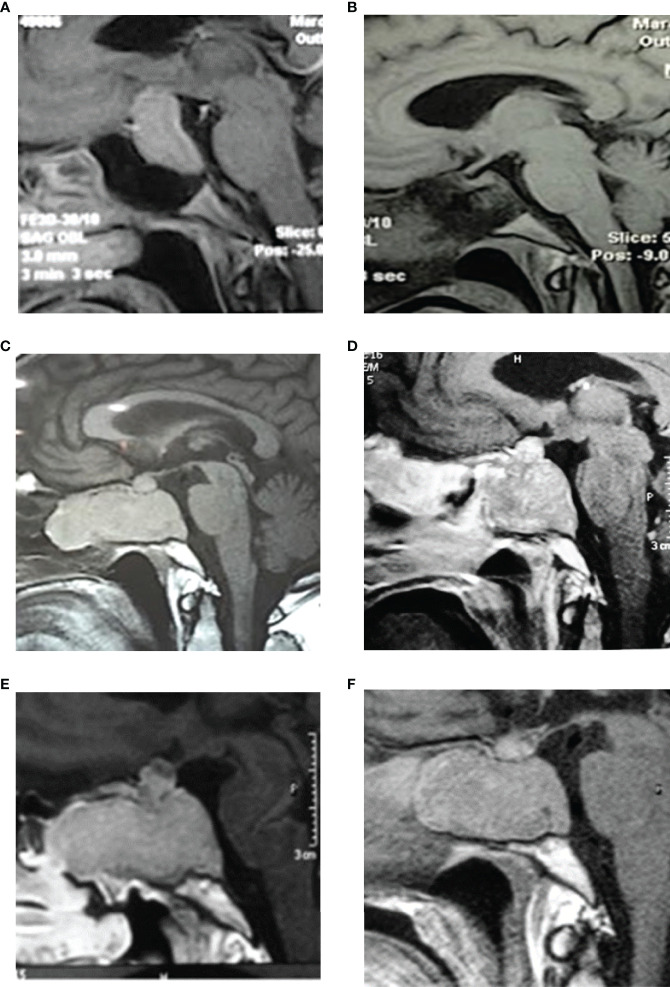
MRI images. **(A)** 2008-Preoperative pituitary MRI with gadolinium (sagittal view), evidencing a large pituitary mass with suprasellar extension and compression of the optic chiasm. **(B)** Postoperative MRI with gadolinium (sagittal view) showing complete tumor resection and decompression of the optic chiasm. **(C)** 2014-pituitary MRI (sagittal view) evidencing a tumor mass with extensive sphenoid and nasal invasion. **(D)** Postoperative MRI with gadolinium (sagittal image) showing partial resection with a remnant tumor in the nasal, sphenoid, and sellar region. **(E)** Control MRI in 2015 shows tumor regrowth in the sagittal image. **(F)** Postoperative MRI (sagittal view) showing less remnant tumor mass than in 2015. Tumor remains stable after radiotherapy and both cabergoline and ketoconazole treatment until 2021.

The patient underwent transsphenoidal surgery with total tumor resection (2008, 1^st^ TSS, **Figure 1** and **Figures 2A, B**). The postoperative medical controls showed visual acuity and visual field recovery. Laboratory studies revealed the need for adrenal hormone substitution for three months while long-term follow-up showed the need for permanent thyroid hormone substitution. MRI controls in the following years showed normal chiasm location; together with visual acuity and visual field unchanged during the period 2009-2012.

The patient left treatment and medical controls until 2014 when she presented in the service with increased body weight, centripetal obesity, high blood pressure, and diabetic symptoms. Endocrine studies showed high plasma cortisol and ACTH levels and high free urinary cortisol characterizing Cushing’s disease (**Table 1**).

The MRI showed a giant tumor mass in the sellar region with critical nasal invasion that infiltrated both cavernous sinuses and extended around carotid arteries. The patient underwent a second transsphenoidal surgery in 2014 (2^nd^ TSS, **Figure 1**). This time, the tumor had turned fibrotic and compromised critical structures (**Figure 2C**) that made it too complex for resection. **Figure 2D** shows the partially resected tumor. As expected, postoperative endocrine studies reported elevated plasma ACTH concentrations and high cortisol levels (**Table 1**).

Subsequently, the patient started pharmacological treatment with 600 mg/day Ketoconazole and 3 mg/week Cabergoline due to hypercortisolism and tumor growth control, respectively. After the failure of this treatment and the persistence of the aggressive tumor characteristics, it was prescribed 150mg/m^2^ Temozolomide (TMZ) 5 days/month for 3 months. This scheme of treatment stabilized tumor secretion and attenuated Cushing´s disease but it did not reduce tumor growth (tumor size: 29x31x23 mm in control MRI **Table 1**). Consequently, a new surgery was performed in 2015 (3^rd^ TSS, **Figure 1** and **Figures 2E, F**) with partial resection of the tumor mass.

The patient continued under Cabergoline and Ketoconazole treatment, and the stereotaxic conformal radiation therapy on tumor remnant didn’t cure but managed to control tumor growth and Cushing’s disease. The whole clinical approach stabilized tumor growth until 2021 (**Table 1**).

### Pathology

#### 2008 Surgery Specimen

Anatomo-pathological examination of the material obtained from surgery was compatible with a pituitary tumor. It had no signs of necrosis, it was negative for p53 and it had a Ki67 index below 1%. It showed few ACTH immunoreactive (IR) cells and it was negative for any other pituitary hormone. The results were conducted to the final diagnosis of silent corticotrope tumor (**Table 1**).

#### 2014 Surgery Sample

Histopathological examination showed tumor cells consistent with a pituitary chromophobe tumor that strangely did not stain for ACTH in spite of secreting this hormone and the typical Cushing disease features (**Table 1**). No other pituitary hormone was positive. A Ki-67 proliferating index of 9% and p53 positive cells demonstrated the aggressive characteristics of the tumor.

#### 2015 Surgery Material

**Figure 3:**
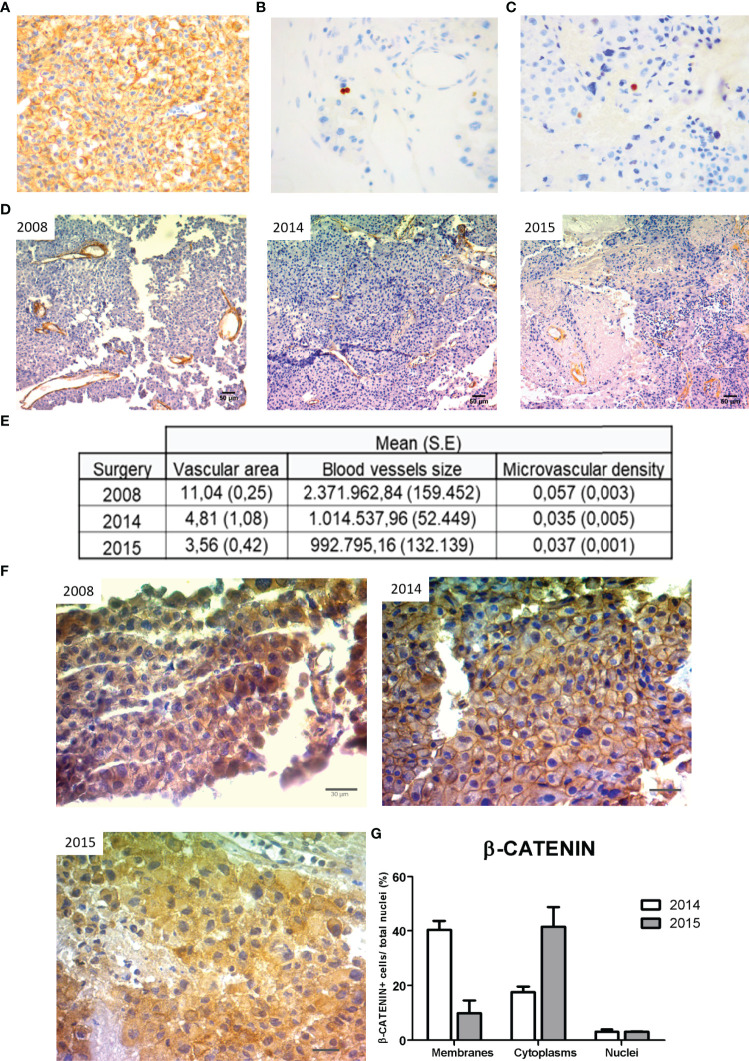
Pathology of the different tumor samples. The excised tumor tissue from the 2015 surgery showed high ACTH IR cells **(A)**, increased Ki-67 proliferation index **(B)** and p53 immunoexpression **(C)** as we determined by IHC (20X, 40X, and 40X magnification, respectively). **(D)** Representative images of immunohistochemistry (IHC) for α-SMA of each tumor specimen obtained in 2008, 2014, and 2015 surgeries, respectively (10X magnification, scale: 50 µm). The vascular area (blood vessels area/total area), blood vessel size (µm2), and microvascular density (number of blood vessels/µm2) determined by α-SMA stained vessels are summarized in the table **(E)**. Values correspond to the mean and standard error of two different experiments of immunohistochemistry. **(F)** Representative images of β-CATENIN immunoexpression in tumor samples of 2008, 2014, and 2015 surgeries, respectively (40X, scale: 30µm). **(G)** β-CATENIN positive cells at membrane, cytoplasm, and nucleus of tumor samples from 2014 and 2015 were counted and expressed as percentages of total nuclei (mean and SE).

The pathology report confirmed in the tumor sample numerous ACTH IR cells when evaluated both by immunohistochemistry (**Figure 3A**), while the tumor was negative for any other pituitary hormone. The tumor proliferation index was 4% (**Figure 3B**), and positive cells for p53 were found (**Figure 3C** and **Table 1**).

### Immunohistochemical Study of α-SMA and β-Catenin Along Aggressive Transformation

We analyzed the expression of the angiogenic marker α-SMA in tumor material obtained from the three surgeries. We determined angiogenic parameters such as the α-SMA blood vessel size, the vascular area, and the microvascular density. Interestingly, the silent corticotropinoma tissue obtained from 2008 surgery denoted the highest angiogenic values. Instead, tumor samples from 2014 and 2015 in which the Ki-67 proliferation index was higher than in 2008, showed a reduction in their vascularization coincident with the aggressive transformation (**Figures 3D, E**).

Then, we evaluated by IHC the expression of β-CATENIN, the main protein of the canonical Wnt cell signaling. Interestingly, we found positive immunostaining for β-CATENIN along pituitary tumor transformation pointing out a possible role of this protein in aggressive corticotroph tumor development and evolution (**Figure 3F**).

This protein is localized in the membrane, cytoplasm, and nucleus of tumor cells (**Figure 3F**), and β-CATENIN quantification was possible in samples from 2014 and 2015 surgeries. Unfortunately, the 2008 tissue was not appropriated for β-CATENIN quantification. We determined a reduction in the percentage of membrane β-CATENIN positive cells in a tumor sample from 2014 to 2015, while cytoplasmic β-CATENIN positive cells showed a marked increase (**Figure 3G**). These results as well as bibliography data led us to hypothesize that β-CATENIN relocalization along with tumor expansion, probably associated with the canonical Wnt pathway activation, could be accompanying tumor aggressiveness as well as contributing to the resistance to TMZ treatment.

Moreover, TMZ chemotherapeutic treatment could be affecting tumor cell proliferation but not ACTH secretion (see **Table 1**), which could be associated in turn, with β-CATENIN persistence and activation.

To summarize, in 2008, the patient was diagnosed with a large silent (without biological and clinical signs of hypercorticism) invasive non-proliferative corticotroph tumor. The first surgery material showed few ACTH IR cells, hypervascularization, and high expression of α-SMA. Six years later (2014), the tumor grew and progressed to a clinically aggressive and secreting corticotroph tumor. The second surgery sample revealed diminution of the vascularization and high expression of membrane β-Catenin. Treatment by temozolomide and third surgery in 2015 managed to restrain the ACTH secretion and the clinical signs. In the tumor, there were found numerous ACTH IR cells, no change in the vascularization, but internalization of β-Catenin. Post-surgery radiotherapy and cabergoline together with Ketoconazole stabilized the Cushing’s disease.

## Discussion

Corticotroph tumors represent the predominant tumor subtype in cohorts of aggressive pituitary tumors and pituitary carcinomas (18). Moreover, it is known that silent corticotroph tumors may turn into aggressive tumors or carcinomas (19, 20). The peculiarities of silent corticotroph adenomas such as their high frequency of recurrence and their invasive capability led the research community to delve into the causes that make them such complex entities to handle (5, 8). Despite the multiple efforts, many questions remain unsolved, as to what mechanisms are driving their “silence” in the clinic, what turns them hormonally active in some cases, which cell markers could predict their evolution to aggressive behavior, or which could be indicative of a better response to treatments, among others.

Here we present a rare case of a patient with a silent corticotroph tumor that compromised the optic chiasm, which recurred as a giant, aggressive ACTH-secreting tumor with a high proliferation index and drug resistance. We describe the course of the lesion and analyzed the histopathological changes in samples of the initial, the relapsed, and the re-grown tumors.

During its transformation, the tumor turned from clinically silent (with no signs of Cushing disease) with a 1% Ki-67 proliferation index in 2008, to Cushing’s disease caused by a giant tumor, with high proliferation rates (9% and 4% in 2014 and 2015, respectively). Contrarily to this case, Moshkin et al. described a silent corticotroph tumor, which also turned recurrent and resisted conventional therapies, with no Ki-67 increments during tumor transformation (21).

The secreting tumor we present here expressed p53 and showed an apparent resistance to 3 months of TMZ treatment in 2014 and 2015 as it was already reported in the literature for ACTH secreting tumors (22).

Several markers, including different cell signaling components, have been investigated to reveal their implication in functioning and/or silent corticotroph pituitary adenomas (23–25). Aberrant activation of Wnt/β-Catenin signaling has been reported in many types of cancers (12, 26–29) as well as in pituitary tumors, in which both canonical and non-canonical Wnt pathways have been linked to tumor development (29–32). Moreover, mutations in the third exon of the β-Catenin gene (CTNNB1), which encodes the GSK3-β binding site and is the degradation-targeting box of β-Catenin, are infrequent in pituitary tumors (33, 34). Importantly, the knockdown of β-Catenin inhibited pituitary adenoma cell proliferation and migration probably by inhibiting AKT, STAT3, Cyclin D1, CDK4, MMP-2, and MMP-9. It suggests that β-Catenin may regulate various downstream molecules to enhance pituitary tumor cell proliferation and invasion (35).

Additionally, Wnt pathway inhibitor WIF-1 was found underexpressed in human bromocriptine-resistant prolactinomas (36) as well as in non-functioning pituitary tumors (37).

β-Catenin is known to play a role in cell-to-cell junctions by associating with *α*-catenin and E-cadherin in the membrane. Several mediators were described to dissociate *β*-catenin from adherens junctions and induce its translocation into the nucleus and activation of Wnt target genes (38, 39).

In our previous work in a cohort of human resistant prolactinomas, we described β-CATENIN expression that localized in membrane, cytoplasm, and nucleus of prolactinoma cells, and we showed a markedly decrease of this key protein in membranes when compared with normal pituitaries, which would indicate its activation (40).

Nevertheless, if we focus the search on the particular group of silent and aggressive corticotroph tumors, available data related to Wnt components’ expression or β-CATENIN relocalization are scarce (25). Indeed, molecular studies on different stages of this kind of pituitary tumor transformation are not commonly found in the literature.

In the current study, we quantified β-CATENIN expression in the samples from 2014 and 2015, which suggested, as we have already reported for resistant prolactinomas (40, 41), a β-CATENIN activation in corticotroph tumor aggressiveness. In this sense, Semba and colleagues also showed nuclear localization of β-CATENIN in half of the pituitary samples of the cohort they analyzed, which included a silent corticotroph adenoma. Normal glands in turn, only showed membrane β-CATENIN expression in that cohort (42).

Additionally to β-CATENIN relocalization, our present results suggest a remodeling of the vasculature across the aggressive transformation. Angiogenesis, a widely studied process by us and by other authors in pituitary pathogenesis (17, 43–48), can affect tumor promotion and therapy responses. Markers of angiogenesis as VEGF and vascular density were reported to increase in aggressive pituitary tumors, although their significance related to response to anti-angiogenic therapy is uncertain (49). In our present study, the evaluation of angiogenic parameters showed that the lesion evolved from a highly vascularized and less proliferative to an aggressive and less vascularized tumor, denoting that aggressiveness was driving tumor growth despite less vascularization. Contrarily, it was reported that microvascular density and VEGF expression were higher in pituitary carcinomas than in benign adenomas (50). In lactotroph tumors, indeed, tumor aggressiveness has been associated with neoangiogenesis, and with Endocan and VEGF overexpression among other factors (51). Similarly, in the case presented by Moshkin et al, with several surgeries as our case, a markedly vascular tumor with dilated, irregular capillaries and moderate VEGF immunostaining in almost all tumor cells was described. Nevertheless, other authors published a reduction in the microvascular density of NF and GH-secreting tumors (52) as well as in some pituitary tumor groups which included ACTH secreting tumors when compared to normal pituitaries (53). Potentially, the reduction in the vascularization that accompanied this tumor transformation could be the result of the fibrotic process found during the second surgery (clinical presentation) and may be mediated by TGF-β1, a stromal fibroblast activator and inhibitor of angiogenesis (54). Additionally, prolonged cabergoline treatment, known to induce fibrosis in tumors and decrease angiogenesis (45), might have also contributed to vascular area reduction (55).

It has been suggested that type I silent corticotroph adenomas have lower levels of MGMT than clinically active ACTH-secreting tumors (56), then, they could be good candidates for TMZ treatment. Contrarily, several works revealed a lack in the response to the drug in this type of pituitary tumor and in more aggressive cases (22, 57). According to a meta-analysis of all reported cases until December 2020, it was stated that functioning tumors respond better to TMZ than non-functioning ones (58).

Our patient received TMZ together with Cabergoline and Ketoconazole. This combination might have attenuated Cushing´s disease, stabilized tumor secretion, and partially decreased cell proliferation index from 9 to 4%, but it did not reduce tumor growth completely. To the contrary, the tumor regrew later. Given the undetectable levels of expression of MGMT in the ACTH secreting tumor (evaluated in 2014 and 2015 lesions), MGMT would not contribute to TMZ resistance and it would probably be the short TMZ cycle (only 3 months) the key point of tumor regrowth from the highly proliferative remnant.

Interestingly, Wnt/β-Catenin has been implicated in the chemoresistance of different types of cancer (15, 59, 60). In particular, its inhibition augments the cytotoxicity of the alkylating agent Temozolomide (TMZ) in colon carcinoma, glioblastoma, medulloblastoma, and neuroblastoma cell lines (61). Nevertheless, as far as we are concerned, the role of β-Catenin in silent-to-aggressive pituitary tumor progression under TMZ treatment has not been investigated yet. In the light of the present case, it seems that β-Catenin activation can contribute to pituitary tumor resistance to TMZ.

We could hypothesize that the activation and persistence of β-CATENIN we found herein is driving the pro tumoral processes, such as proliferation probably mediated by Cyclin D1 (recently demonstrated by us in *in vitro* models of prolactinomas 40), as well as disorganization of tumor vasculature and the increase in hormonal secretion. In turn, it could be indicating, that β-CATENIN activation might be driving tumor TMZ resistance.

Finally, *in vivo* and *in vitro* studies that explain Wnt signaling and active β-CATENIN participation in aggressive corticotroph tumor progression as well as the response to treatments could provide complementary information to consider Wnt signaling as a possible new therapeutic target for aggressive ACTH producing pituitary tumors.

## Data Availability Statement

The datasets for this article are not publicly available due to concerns regarding participant/patient anonymity. Requests to access the datasets should be directed to the corresponding author.

## Ethics Statement

The study was approved by Comité de Ética en la Investigación y en uso de animales de experimentación de la Universidad Nacional del Noroeste de la Provincia de Buenos Aires (COENOBA). The patient provided the written informed consent to participate in the study.

## Author Contributions

GD performed experimental analyses, organized data, and wrote the first manuscript. SP contributed with experimental data quantification. GER, CDB, JPC and GS provided clinical data, imaging, and important clinical expertise. SB managed the case, performed the surgeries, highlighted the particularity of the case, and gave invaluable clinical information. CC organized the group and data, designed the work, and wrote the final version of the paper. All authors contributed to manuscript construction and revision and approved the final manuscript.

## Funding

We thank the following institutions that financially supported our work, Agencia Nacional de Promoción Científica y Tecnológica, Argentina [PICT 901-2013], Consejo Nacional de Investigaciones Científicas y Técnicas and Universidad Nacional del Noroeste de la Provincia de Buenos Aires [PIO CONICET-UNNOBA 2015-2016 15720150100010CO], and SIB UNNOBA [#0241/2017]. The three grants were awarded to CC.

## Conflict of Interest

The authors declare that the research was conducted in the absence of any commercial or financial relationships that could be construed as a potential conflict of interest.

## Publisher’s Note

All claims expressed in this article are solely those of the authors and do not necessarily represent those of their affiliated organizations, or those of the publisher, the editors and the reviewers. Any product that may be evaluated in this article, or claim that may be made by its manufacturer, is not guaranteed or endorsed by the publisher.
